# Driver Speed Profiles Index Warning Signs of MCI

**DOI:** 10.1093/geroni/igab046.489

**Published:** 2021-12-17

**Authors:** Jennifer Merickel, Ruiqian Wu, Matthew Rizzo, Ying Zhang

**Affiliations:** 1 University Of Nebraska Medical Center, Omaha, Nebraska, United States; 2 University of Nebraska Medical Center, Omaha, Nebraska, United States

## Abstract

**Goal:**

Use driver behavior profiles to screen and index early warnings of cognitive decline and Alzheimer’s disease (AD). Hypothesis: Real-world driver speed behavior profiles discriminate mild cognitive impairment (MCI).

**Methods:**

Sensors were installed in personal vehicles of 74 legally-licensed, active drivers (age: 65-90 years, μ = 75.85) who completed 2, 3-month real-world driving assessments, including demographic and cognitive assessments, 1 year apart (244,564 miles driven). MCI status was indexed using 8 neuropsychological tests (spanning executive function, visuospatial skills, processing speed, and memory), relevant to MCI and driving. Driving environment was indexed from state speed limit (SL; roadway type: residential, commercial, interstate) and sunrise-sunset databases (time of day: day vs. night). Models: Data were randomly split into training (66%) and validation (33%) sets. An optimal mixed effects logistic regression model was determined from validation data AUC values.

**Results:**

MCI drivers drove slower with optimal discrimination (estimated for every 5 mph decrease in speeding) in 1) residential roads (SL 25-35 mph; MCI odds increased by 6% [95% CI: 2-11%]), 2) interstate roads (SL >55 mph; MCI odds increased by 14% [95% CI: 8-20%]), and 3) night environments (MCI odds increased by 7% [95% CI: 2-12%]).

**Conclusion:**

Quantitative indices of real-world driver data provide “ground truth” for screening and indexing phenotypes of cognitive decline, in line with ongoing efforts to link driver behavior with age-related cognitive decline and AD biomarkers. Behavioral biomarkers for diagnosing early warnings of dementia could ultimately bolster our ability to detect and intervene in early AD.

